# Describing movement learning using metric learning

**DOI:** 10.1371/journal.pone.0272509

**Published:** 2023-02-03

**Authors:** Antoine Loriette, Wanyu Liu, Frédéric Bevilacqua, Baptiste Caramiaux

**Affiliations:** 1 STMS IRCAM-CNRS-Sorbonne Université, Paris, France; 2 Sorbonne Université, CNRS, ISIR, Paris, France; University of Innsbruck, AUSTRIA

## Abstract

Analysing movement learning can rely on human evaluation, e.g. annotating video recordings, or on computing means in applying metrics on behavioural data. However, it remains challenging to relate human perception of movement similarity to computational measures that aim at modelling such similarity. In this paper, we propose a metric learning method bridging the gap between human ratings of movement similarity in a motor learning task and computational metric evaluation on the same task. It applies metric learning on a Dynamic Time Warping algorithm to derive an optimal set of movement features that best explain human ratings. We evaluated this method on an existing movement dataset, which comprises videos of participants practising a complex gesture sequence toward a target template, as well as the collected data that describes the movements. We show that it is possible to establish a linear relationship between human ratings and our learned computational metric. This learned metric can be used to describe the most salient temporal moments implicitly used by annotators, as well as movement parameters that correlate with motor improvements in the dataset. We conclude with possibilities to generalise this method for designing computational tools dedicated to movement annotation and evaluation of skill learning.

## Introduction

Motor skill learning is defined as the ability to perform a movement better, according to some given criteria such as speed or accuracy, in comparison to a reference movement [1]. Metrics used to assess motor learning usually rely on error-rates or movement variability measures. However, such measures do not necessarily reflect the way humans perceive movement improvements: people might instead focus on specific movement features or agglomerate several criteria established qualitatively. Our long term goal is to establish metrics that could describe human perception of movement improvement during motor learning or adaptation processes. In this paper, we propose a method utilising *metric learning* in order to describe human ratings of motor improvement.

Metric learning is a machine learning technique that aims at finding the best distance function between datapoints so as to optimise a cost function. For example, a Mahalanobis distance can be learned in the context of a classification task to maximise the score of a k-nearest neighbour classifier [2]. Learned metrics typically improve performance in various machine learning tasks (classification or clustering among others). Several metric learning surveys have been published presenting the general approach [3, 4] as well as focusing on deep learning [5]. Interestingly, metric learning can also be used as an analysis tool in relation to human annotated databases. For instance, the perception of musical instrument timbre was investigated by analysing the structure of learned metrics from ratings of sounds similarity [6].

For body pose and movement perception, metric learning was first applied with human comparison of still images from datasets containing skeleton data. Harada et al. [7] optimised the correlation between a weighted sum of joint distances and human ratings to show that wrist, neck and head were the most important joints for explaining body pose similarity. A similar method was investigated by Tang et al. [8]. In both cases, the relative values of the optimised weights reflected the importance of their associated joints for human perception of body pose. Marinoiu et al. [9] derived a metric from data (using Relevant Component Analysis [10]) using the way humans reproduced poses they had seen on videos before analysing how it differed from standard Euclidean distance. As movement is dynamic, previous work also looked at ways to take movement temporal structure into account. To that extent, Ofli et al. [11] used a measure based on information theory and variance analysis to investigated which joints were the most informative at specific times in videos for action recognition. Krüger et al. [12] explored the effect of different input features on the correlation of a Dynamic Time Warping (DTW) metric (representing the cost of temporal alignment between two examples), with similarity ratings produced by humans based on videos. Finally, combining Mahalanobis distance learning and temporal alignment using Dynamic Time Warping has been investigated to improve the performance of classification of handwritten signatures [13], or more generically multivariate time series [14].

To our knowledge, metric learning has not yet been investigated in the context motor learning. In a recent study, Le Naour [15] showed that expert ratings of gymnastic movements did not match measures obtained from quantitative analysis, calling thus for novel methods able to derive metrics based on human ratings.

In this paper we investigate whether a computational metric can be learnt from human rating in a context of motor learning. Precisely, we propose to learn DTW-based distances on human movement such as maximising the correlation with human rating. Our objective is then to interpret the learned metrics for motor learning analysis. To do so, we employ a dataset that was initially collected to study how users learn long gesture sequences from videos, over several days. Each participant was asked to practice the movement sequence to be as close as possible to a video reference. By rating this dataset, we can then use a metric learning approach taking into account examples that are considered as similar [16, 17]. Our contributions are threefold. First, we confirm that there is a correlation between the human rating and the DTW metric, which we denote the “baseline correlation”. Second, we seek to optimise this baseline correlation and examine whether such procedure allows us to estimate the most prominent parameters used by the raters. We consider two types of parameters. The first one investigates the movement features (position, velocity, acceleration, amongst others) of movement execution; the other explores temporal segments, i.e. focus of attention over time. Third, we examine how these parameters used by the raters are implied by motor learning processes for our specific case.

The paper is structured as follows. We introduce the method we propose, including the dataset we annotated. Then, we report on the results and discuss them.

## Method

### General approach

Our goal is to train a similarity metric between movements which matches perceived similarity expressed by human ratings. Human annotation of perceived movement similarity is challenging for several reasons. First, it might be difficult to establish objective criteria for rating [18]. Second, absolute continuous ratings are prone to large discrepancy between raters. To avoid this, a better strategy is to ask raters to compare pairs of movements [16], or to compare two pairs relative to a reference movement [17]. This is a well-documented finding which recommends relative over absolute judgement or assessment [19, 20]. This strategy is adopted in the present paper, which we call *relative* movement similarity assessment.

**Fig 1:**
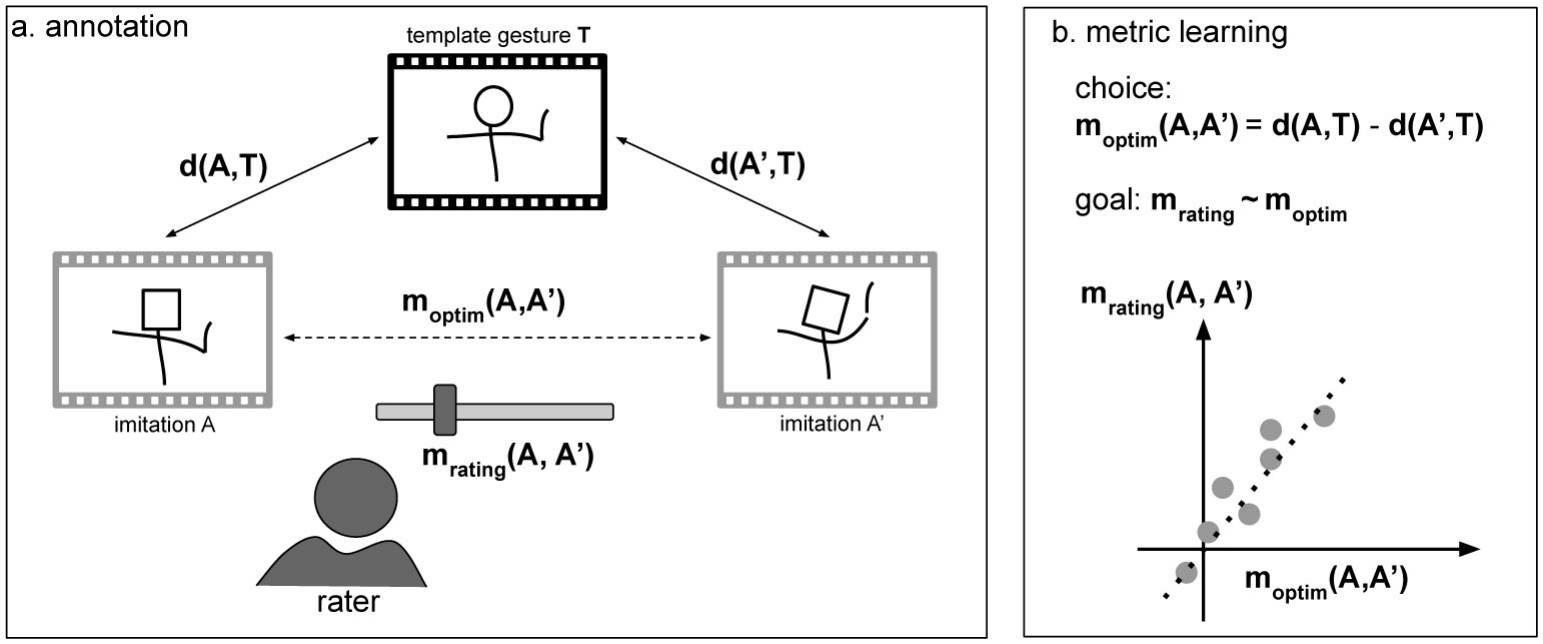
Overall setup for the paper. (a) Performance pairs (*A*, *A*′) are sampled for comparison against a reference *T* by judges on videos and computing algorithms on sensor data. (b) Metric learning acts on parameters of the compute function *m*_*optim*_ through search or optimisation to produce a meaningful relationship with *m*_*rating*_ through a distance function *d*.

Fig 1 depicts the general approach. We consider the comparison between two video-tapped movements of a person learning to perform a template gesture. First, we collect human ratings on a given set of recorded performed movements, and second, we learn a computational metric that matches, as close as possible, these human ratings.

In the first step, a movement database containing several performances of a given movement template is chosen and rated (Fig 1(a)). Precisely, raters give, for a pair of movements *A* and *A*′, a value between −1 and 1 expressing how close each movement *A* and *A*′ is to a template *T*: −1 corresponding to *A* being the closest to *T*, 1 corresponding to *A*′ being the closest to *T*, and 0 where *A* and *A*′ being judged as equally close to template *T*. In the second step, we compute a *parametric* similarity metric between movement *A* (resp. *A*′) and template *T* (resp. *T*). The difference between the two metric results is compared to the human relative similarity metric for each corresponding pair. By acting on the metric’s parameters, we can optimise the correlation between the *parametric* similarity metric and the human similarity judgement, assuming that the relationship between DTW differences and ratings is linear.

The following subsections detail the annotation and learning procedure that were developed.

### Dataset

**Fig 2:**
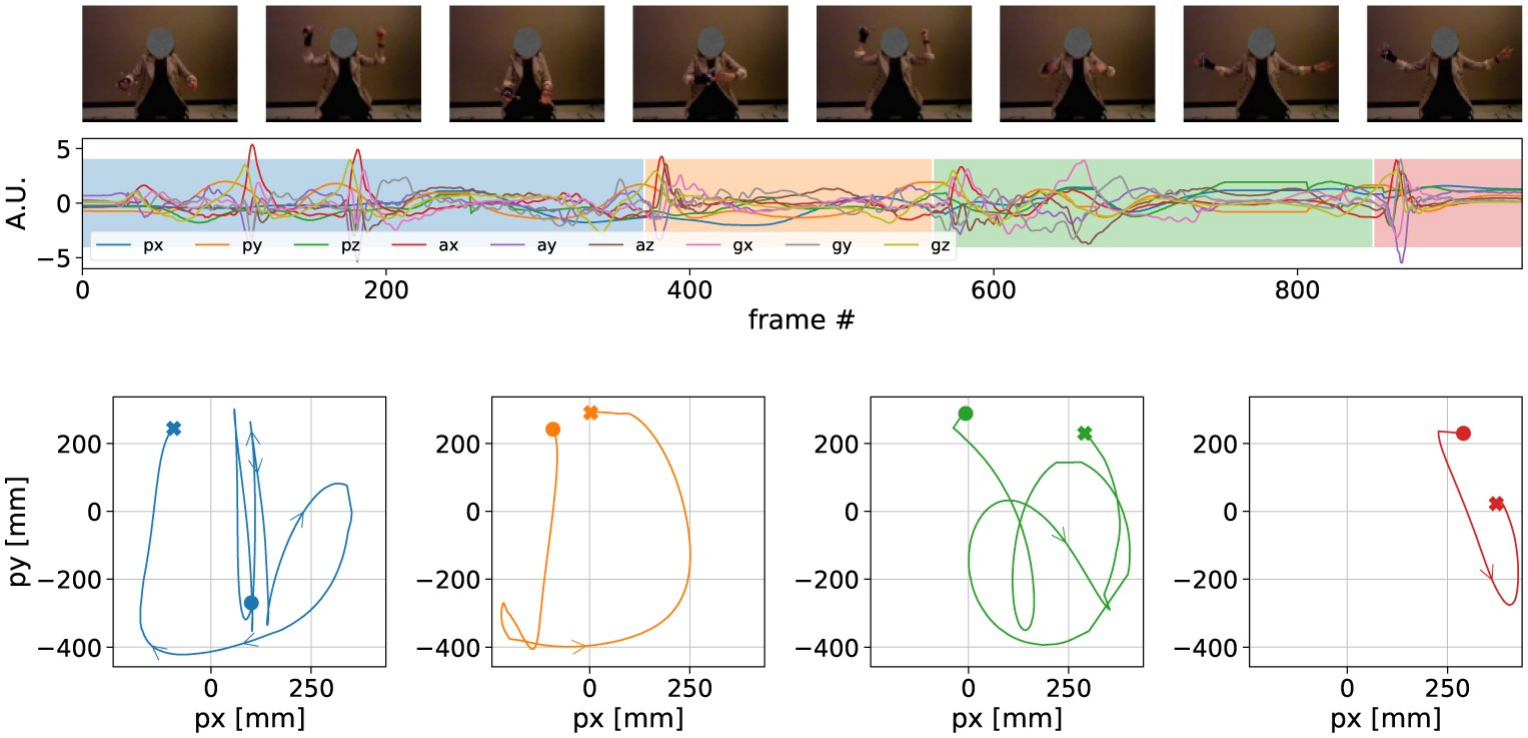
Stills from video (top), times series from sensor data (middle) and position data (bottom) for the template gesture, segmented in the the four phrases. In the middle graph, the short peaks in the time series correspond to vertical strokes that can be seen in the video. The position (x, y) provided by the Optitrack sensor is seen on bottom graphs. For each phrase, the starting point is marked by a circle while the last sample is indicated with a cross. The colours are matching between the sensor time series and the segmented position data.

We used a publicly available dataset, previously used in a motor learning study [21] where it was investigated how participants learned from video a complex hand movement, referred to as template gesture in the following. This template gesture can be schematically represented by a sequence of four phrases (Fig 2, bottom panel). It was designed with a variety of ‘strokes’ and specific spatial patterns, reminiscent of conducting gestures. For instance, vertical strokes followed horizontal inwards and outwards movements, as visible in Fig 2, top panel. The video of the template gesture is provided in the supplementary materials.

The template gesture was performed by the person who designed the gesture, seated on a chair, wearing on the right hand a custom-made glove equipped with optical markers and an IMU (Inertial Measurement Units), while being filmed (Fig 2, top panel). The gesture was specifically designed to be performed with the hand, so most of the information was indeed carried by the hand. A total of 24 participants were asked to learn this template gesture over three sessions occurring during three different days in a week, under various conditions. In this article, we selected the dataset related to 12 participants having learned the template gesture in the condition which did not involve audio feedback. During each session, participants were equipped similarly in addition to having the template gesture video shown to them. Thus, the same movement data and video recording are available for the participants’ performances and for the template gesture. Precisely, motion capture data (3d positions (*x*, *y*, *z*)) and inertial data (3D accelerometers and 3D gyroscopes) are available at a sampling rate of 100Hz, as shown in Fig 2, middle panel. The video was captured at 30fps. For more details on the experimental protocol used in the dataset collection, please refer to [21].

### Human annotation

The annotation task consisted in providing a continuous measure assessing which video of a pair of movements (A, A’) showed the movement most similar to the video of the gesture template.

#### Annotation tool

For this, we designed an interface displaying the video of the template gesture above the videos of the current pair (*A*, *A*′) to evaluate (see Fig 8 in S1 Apparatus). Between videos of *A* and *A*′, a slider, which default position was centred, allowed raters to set their rating. The left and right positions of the slider were associated to the video on the left and right, respectively.

#### Dataset sampling

We considered several strategies for creating the pairs of movement. After several pretests, we chose that each pair of videos A and A’ were to be performed by the same participants, but related to different learning sessions (i.e. different days). In that case, we hypothesised that the movement differences recorded in videos A and A’ would be noticeable. Thus, to select each given pair, a participant was first randomly chosen (in a total of 12), and two performances were randomly chosen provided they belonged to different sessions (days).

#### Annotation datasets

Three different annotation datasets were created, due to practical limits. First, a dataset *D*_*inter*_, consisting of 90 video pairs, was annotated by all four paper authors. This allows us to test the raters agreement. Second, the annotation dataset *D*_*intra*_ was built to evaluate the intrarater reliability. For this, the first author re-annotated 45 video pairs, from the previous annotation dataset, twice at a month interval. Third, a annotation dataset *D*_*single*_ which contained 180 video pairs annotated by a single rater (each author annotated 45 pairs). As described later, this annotation dataset provided additional data feeding the metric learning, while its consistency can be evaluated.

### DTW-based metric learning

The learning goal is to optimise the parameters of a movement similarity metric based on human ratings. We propose to use a DTW-based metric (Dynamic Time Warping [22]). DTW allows for handling temporal structure of the movements and remains one of the most used metric for time series analysis, in addition to being easily parameterisable and interpretable. Formally, the DTW between two time series **X** and **Y**, of sizes *N* and *M* respectively, is the sum of element-wise distances over the optimal path *p*, where *p* = {(*n*, *m*)_*k*_, *k* ≥ *min*(*N*, *M*)}:

$$\begin{array}{c} DTW\left(X,Y\right)=\sum_{i\in p}\left|\right|x_{i(0)}-y_{i(1)}{\left|\right|}_{1} \end{array}$$ (1)

where **x**_*i*(0)_ (resp. **y**_*i*(1)_) is the feature vector of movement **X** (resp. **Y**) at time *i*(0) (resp. *i*(1)), where *i*(0) (resp. *i*(1)) is the first (resp. second) index of the element *i* in path *p*, and where ||**x**||_1_ is the L1-norm.

We propose to investigate weighted versions of the DTW acting on either feature dimension or temporal dimension. The adjustment of these weights should ideally reproduce the weights implicitly used by raters in judging the similarity between movements.

In the following, a movement *A* is represented as a multidimensional time series **A** of length *N*, where the feature vector at time *i* is written $a_{i}=\left(a_{i}^{0},a_{i}^{1},\ldots ,a_{i}^{K}\right)$ of dimension K. For instance, **a**_*i*_ can be a vector made of movement position, velocity, acceleration along the three dimensions (*x*, *y*, *z*), leading in this case to *K* = 9. We present the two weighted versions of DTW for the task of aligning movement **A** onto template **T**.

#### Weighting the movement features

The first version of the weighted DTW is based on feature weighting. The goal is to adapt some weights on movement features, such as positions, velocities, accelerations and/or IMU-based features acceleration including gravity, and angular velocities. For two movement time series, such as template **T** and movement **A**, we denote this distance *DTW*_*feature*_(**T**, **A**), which is defined as:

$$\begin{array}{c} DTW_{feature}\left(T,A\right)=\sum_{i\in p}\sum_{k=1}^{K}w_{k}\left|\right|t_{i(0)}^{k}-a_{i(1)}^{k}{\left|\right|}_{1} \end{array}$$ (2)

where $t_{i(0)}^{k}$ (resp. $a_{i(1)}^{k}$) is *k*-th dimension of feature vector **t** (resp. **a**) at time *i*(0) (resp. *i*(1)) *w*_*k*_ is the weight on feature dimension *k*. The weights (*w*_*k*_)_*k*_ are to be optimised. We imposed two constraints: *w*_*k*_ > 0, ∀*k* and a regularisation with $\frac{1}{K}\sum_{k}w_{k}=1$.

#### Weighting the temporal segments

We also propose to weight different time segments of the movement sequence with respect to the template gesture. In this case, optimising the metric corresponds to adapting the relative importance of each segment. Considering two movements such as template **T** and movement **A**, we first compute standard DTW distances between these movements, which produce temporal alignment path between **A** and **T**. The optimal path *p* is then segmented in *N* segments *p*_*i*_ of equal size with regards to the unaligned indices of the template gesture. The segments *p*_*i*_ are only defined with regards to the template gesture, which make this operation asymmetrical, but ensures that meaningful comparisons can be made on the same segments between different aligned movements **A** and **A**′. We denote this distance *DTW*_*segment*_(**T**, **A**), which is defined as:

$$\begin{array}{c} DTW_{segment}\left(T,A\right)=\sum_{j=1}^{N}w_{j}\sum_{i\in p_{j}}\left|\right|t_{i(0)}-a_{i(1)}{\left|\right|}_{1} \end{array}$$ (3)

where (*w*_*j*_)_*j*_ are the weights to be optimised. Here again, we imposed the constraints: *w*_*j*_ > 0, ∀*j* and ∑_*j*_ *w*_*j*_ = 1.

#### Defining the cost function for learning weights

Given ratings *m*_*rating*_(**A**, **A**′) reflecting the perceived relative similarity of *A* compared to *A*′ with respect to a given target gesture *T*, we seek to optimise the DTW metric:

$$\begin{array}{c} m_{optim}\left(A,A^{\prime }\right)=DTW_{optim}\left(T,A\right)-DTW_{optim}\left(T,A^{\prime }\right) \end{array}$$

maximising the Pearson correlation between the *m*_*optim*_ and *m*_*rating*_ for all sampled pairs of movements *A*, *A*′, where *m*_*optim*_ refers to either *m*_*feature*_ (involving *DTW*_*feature*_) or *m*_*segment*_ (involving *DTW*_*segment*_).

#### Implementation and optimisation strategy

In our implementation, we used an open-sourced version (https://github.com/slaypni/fastdtw) of the DTW algorithm [23] modified to support the computation of fast Mahalanobis based distances. The DTW radius was set to 10, after preliminary testing, to balance accuracy and compute time.

The optimisation is performed using the L-BFGS-B algorithm [24], which is a particularly efficient algorithm for optimisation, useful when the number of datapoints is small, as compared to popular gradient based approaches. The loss function is defined as the Mean Square Error (MSE) between the generated distance and the annotation value. Once trained, we report in the result the Pearson’s correlation between the vector of ratings and the vector of learned distances. The optimisation is ran until convergence of the loss, defined as a relative change between two steps smaller than 1*e*^−3^.

The cross validation used a repeated K-fold procedure (K = 2, 8 repeats) over datasets *D*_*inter*_ and *D*_*single*_, splitting each time in half the 12 participants for training and for testing. The 2-folds 8-repetitions procedure provided in total 16 estimates. The performance is evaluated and reported on the testing sets of *D*_*inter*_.

## Results

In this section we present our main findings. More precisely, we validate our datasets through the analysis of annotation reliability between raters. Then we compute the correlation between ratings and the standard DTW metric. This allows us to establish a baseline value that we compare to both learning cases: feature-based and segment-based. Finally, we show that the optimised weights can be used for movement learning analysis.

### Annotation reliability

We used the Intraclass Correlation Coefficient (ICC) [25] to measure the degree of agreement between raters, in the *D*_*inter*_ dataset.

**Fig 3:**
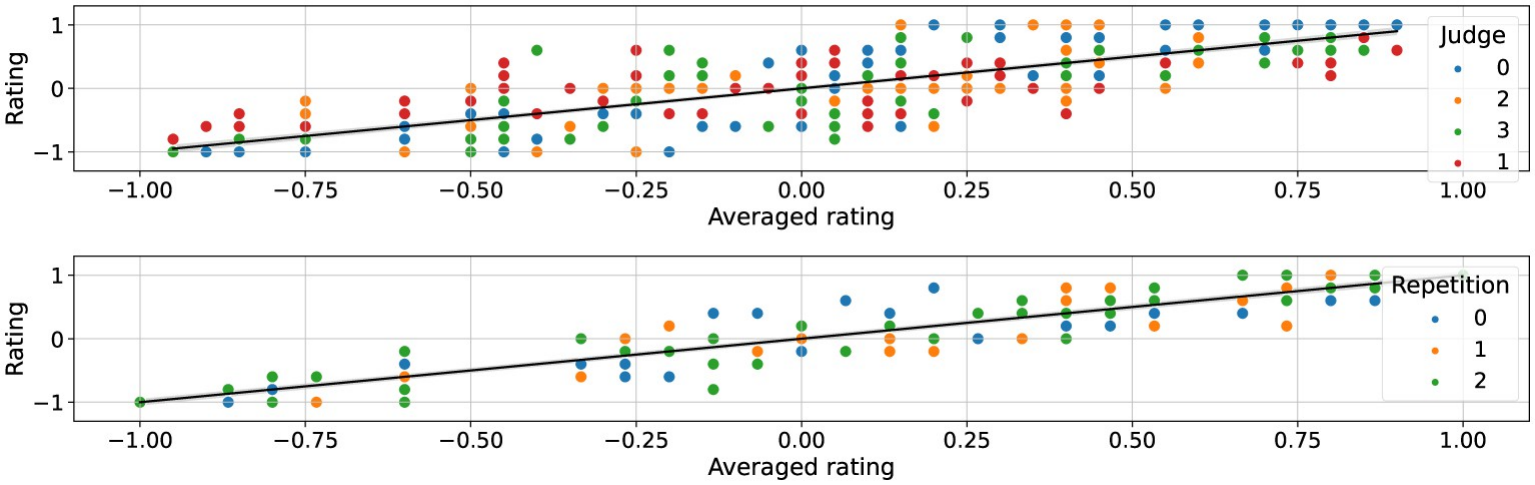
Distribution of ratings against their averaged value for dataset *D*_*inter*_ (top) and *D*_*intra*_ (bottom). The distribution around the averaged value is representative of the annotation noise, which appears greater for different raters as compared to different annotation session for the same rater, as reflected by comparing the ICC values of *D*_*inter*_ and *D*_*intra*_.

First, the ratings over *D*_*inter*_ of all four raters are shown in Fig 3 (top). The ICC estimate based on a single-rating (*k* = 1), absolute-agreement, 2-way mixed-effects model is 0.58 with 95% confident interval = 0.49 − 0.68 (*F*_89,267_ = 6.70, *p* < 0.001). Such a value for ICC is considered as ‘good’. If we considered the ICC calculated based on a mean-rating (*k* = 4), absolute-agreement, 2-way mixed-effects model, a value of 0.85 is obtained, with 95% confident interval = 0.79 − 0.90 (*F*_89,267_ = 6.70, *p* < 0.001). Such a value can be considered between good and excellent.

Second, considering the dataset *D*_*intra*_, shown in Fig 3 (bottom), the ICC calculated based on a mean-rating (*k* = 3), absolute-agreement, 2-way mixed-effects model, is 0.93 with 95% confident interval = 0.90–0.96 (*F*_44,88_ = 16.29, *p* < 0.001). Therefore, we found that reliability was higher for this particular rater, with repeated annotation over time, compared to the interrater reliability (*t*(131) = 2.75, *p* < 0.005). This was expected and shows that the interrater differences are likely due to some perceptual differences rather than notators being uncertain when annotating movements.

While these results cannot be generalised to other raters, they are still important for our method since they show that 1) the rater group is consistent enabling us to use the annotation mean 2) additional ratings of this group could be used, even if the movement is annotated by a single rater.

### Establishing a correlation baseline, without optimisation

We question first whether the DTW metric, applied between the template and the movement pairs, correlates with the annotations. We investigated the effect of the feature space (which feature is used in the distance) on this correlation. To do so, we employed a grid-search approach with selected groups of input features.

We consider a feature space of a total of 5 types of features: acc (from the accelerometers), gyr (from the gyroscope), p0 (position from the optical motion capture), and finally p1, p2 being respectively the first and second derivative of p0. Each of these types has three spatial dimensions, denoted (*x*, *y*, *z*), leading to a total of 15 features. p1 and p2 where computed using a Savitzy-Golay filter (window length of 17 samples and third degree polynomial fit). Each parameter was standardised per dimensions (zero mean and unit variance). We tested each 5 types individually, along with three possible combination as reported in Table 1. Correlation coefficients were computed using cross-validation (see Method).

**Table 1:** Pearson correlation coefficient mean and standard deviation across cross-validation splits for different input feature combinations. The two highest coefficients are marked in bold (statistically not different).

sensor data			computed data		correlation	
					*μ*	*σ*
acc	.	.	.	.	0.713	0.058
.	gyr	.	.	.	0.697	0.054
.	.	p0	.	.	0.495	0.061
.	.	.	p1	.	0.687	0.054
.	.	.	.	p2	0.573	0.066
acc	gyr	p0	.	.	**0.753**	0.040
.	.	p0	p1	p2	0.684	0.040
acc	gyr	p0	p1	p2	**0.759**	0.036

We ran an ANOVA on correlation coefficients with the parameter types as independent variable. We found a significant main effect (*F*_7,120_ = 45.77, *p* < .001, $\eta_{p}^{2}=0.72$). Post-hoc tests with Holm corrections highlighted several differences. For individual types, acc, gyr and p1 produced the best (and comparable) correlations at 0.713 (0.058), 0.697 (0.054) and 0.687 (0.054), respectively. Features p0 and p2 produced the lowest (and statistically equivalent) correlations at 0.495 (0.061) and 0.573 (0.066). In other words, the position data was less informative than inertial data (acc or gyr). When combining three parameter types, we found that the combination (acc, gyr, p0) correlated better with ratings than (p0, p1, p2). Overall, using all five feature together produced the best level of correlation at 0.759 (0.036), while the difference with (acc, gyr, p0) was not statistically different.

**Fig 4:**
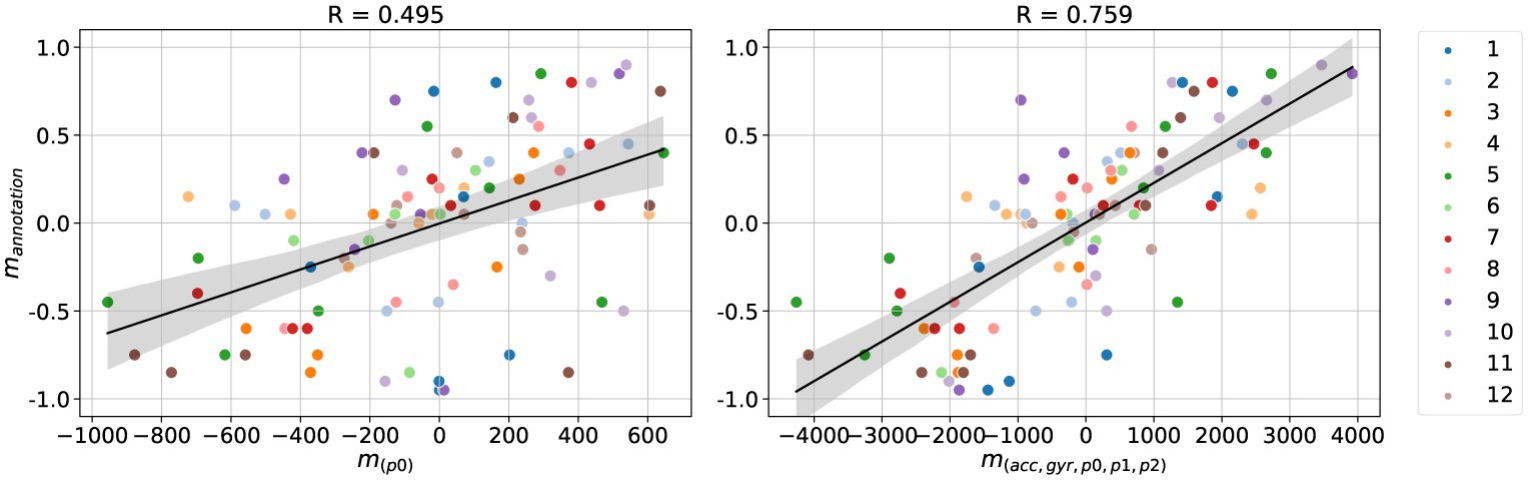
Effect of input features on Pearson correlation. The scatter plots represent dataset *D*_*inter*_ with unique colours associated to participants. The DTW differences against the ratings displayed a stronger linear relationship with all input features included (right) than with just the position (left). These two combinations were chosen as they exhibit the worst and best performance from Table 1.

We further examine the relationship between the computed similarity metric and ratings. Scatterplots of values for ratings (*m*_*rating*_) and similarity metrics based on two sets of features (*m*_(*p*0)_ and *m*_(*acc*, *gyr*, *p*0, *p*1, *p*2)_) are shown in Fig 4, on the left and right, respectively. Comparing the left and right figure furthermore indicates that the improvement of the correlation factor with specific types of features can indeed be explained by lowering the noise level. Visual inspection of scatterplots for different feature combinations did not hint that other models would be more suitable for explaining how these two variables could be related.

In summary, this confirms our assumption that the relationship between the similarity metric derived from DTW differences and ratings can be characterised using the Pearson’s correlation. In the next section, we examine how this correlation can be further optimised.

### Optimising the similarity metric

In this section, we present our results on metric learning. We show that the correlation can be improved through weight learning and, more importantly, that the weights can be interpreted with respect to motor learning.

#### Weighting movement features

We optimised the weights on each dimension used in the DTW distance (denoted *w*_*k*_ in Eq 2) to maximise the correlation with human ratings. The optimisation achieves a mean correlation coefficient of 0.772 (0.040), where the statistics are computed over the cross-validation folds (see Method for more details on the optimisation procedure). This is a slight improvement compared to the results obtained in the previous section using all parameters without weighting (0.759 (0.036)). Nevertheless, paired t-tests confirmed the optimised value is significantly higher to the previously found coefficient (*t*(15) = 2.438, *p* < 0.05).

**Fig 5:**
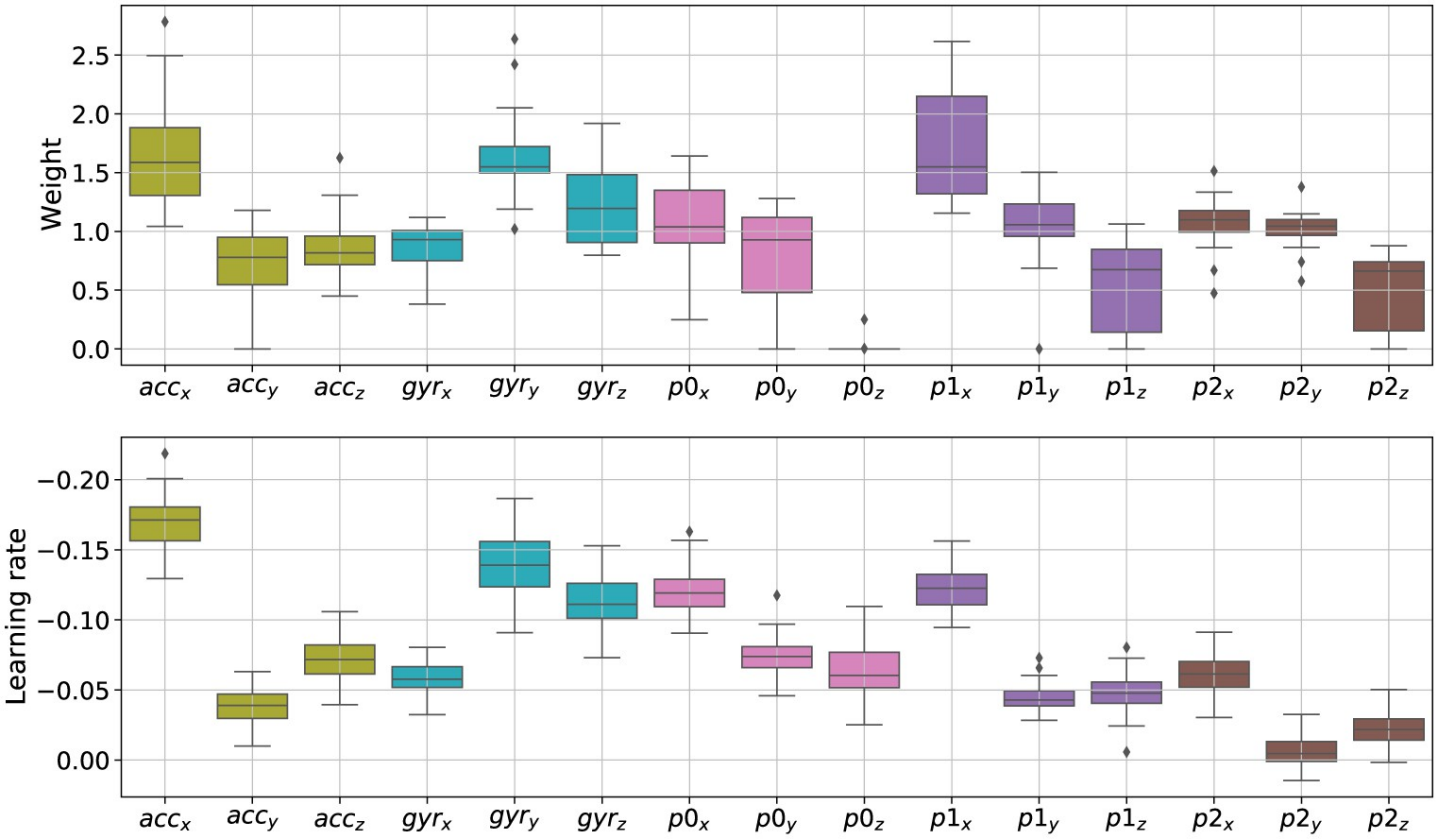
Optimisation weights (top) and learning rate extracted from residual errors (bottom) across all 15 spatial dimensions. Colours are unique per data type (acc, gyr, p0, p1, p2).

The optimised mean weights per dimension are depicted in Fig 5, top panel. We ran an ANOVA on the optimised weight values with Feature as the independent variable. We found a significant main effect (*F*_14,225_ = 29.92, *p* < .001, $\eta_{p}^{2}=0.65$). Post-hoc tests with Holm corrections highlighted several differences. First, the weights are lower on the *z*-axis for the position and its derivatives. This is coherent with the fact that the movement was mostly performed in the (*x*, *y*)–plane. Also, some weights are significantly higher than others within groups of sensors (e.g. *acc*_*x*_ with respect to *acc*_*y*_ and *acc*_*z*_ or *gyr*_*y*_ as compared to *gyr*_*x*_ and *gyr*_*z*_). This suggests that annotators might have used these movement features to find differences within the pair of movements with respect to the template.

Finally, we analysed the relationship between the optimised weights and movement learning. We recall that the ratings were related to performances that happened at different sessions (days) when learning the template. Thus, ratings are expected to reflect participants’ improved performance in imitating the model. To answer this question, we examined whether the movement features that are important to optimise the correlation (given by the weights in Fig 5, top panel) are also the ones that exhibited more improvement. To do so, we computed motor learning rates associated to each movement parameters separately. For this, we used the baseline DTW alignment with all 15 features, between performed movements and the template, and we extracted the errors per feature along the aligned path. Then, we computed motor learning rates by fitting an exponential function on the errors (see DTW errors and learning rates per movement feature, Fig 9 in S1 Apparatus). The learning rates are reported in the Fig 5 bottom panel. Interestingly, there is overall a good correspondence between the learning rates and the optimised weights: a Pearson’s correlation coefficient computed between means reached *r*(15) = −0.62 (*p* < 0.05), which seems to confirm our hypothesis. The higher the weight of a feature, the lower the exponential coefficient, which means a high learning rate.

#### Weighting temporal segments

**Fig 6:**
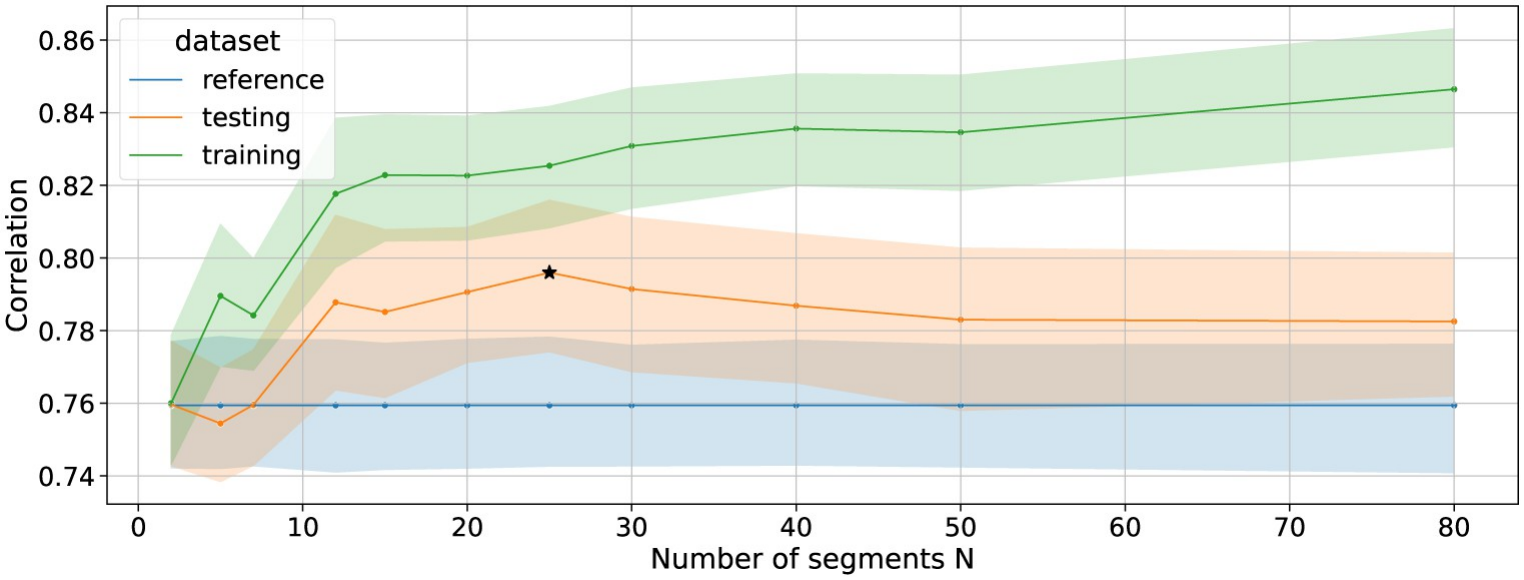
Influence of the number of regions on the correlation for training and testing. The best performance (marked with an asterisk) occurred for *N* = 25, with fewer segments the model was not flexible enough and with more segments the model started to overfit.

Regarding the segment-based optimisation, we iterate on different values of number of segments. We considered 11 choices of the number *N* of segments (i.e. [2, 5, 7, 12, 15, 20, 25, 30, 40, 50, 80]). For each number of segment, we ran the optimisation using cross-validation, similarly as before. In Fig 6, the blue line reports the baseline correlation (cross-validated correlation coefficients when considering the standard DTW on the whole movement, as described when establishing the baseline); the green line reports the correlation values obtained on the training sets while optimising the weights on each segment; the orange line reports the correlation values obtained on the test sets. We found that, for small values of *N*, the model could not learn a meaningful solution and the correlation did not improve. For higher values of *N*, qualitatively between *N* = 20 and *N* = 30, the model seems to be able to learn meaningful weights which improves the correlation. At *N* = 25, a Student’s T-test analysis shows a significant performance improvement against baseline (*t*(15) = 2.549, *p* < 0.05) with a mean correlation coefficient of 0.796 (0.043). For *N* higher than 30, the mean correlation on the test set decreases and is not significantly higher than the mean baseline correlation. This suggests that the model overfits on the training sets.

**Fig 7:**
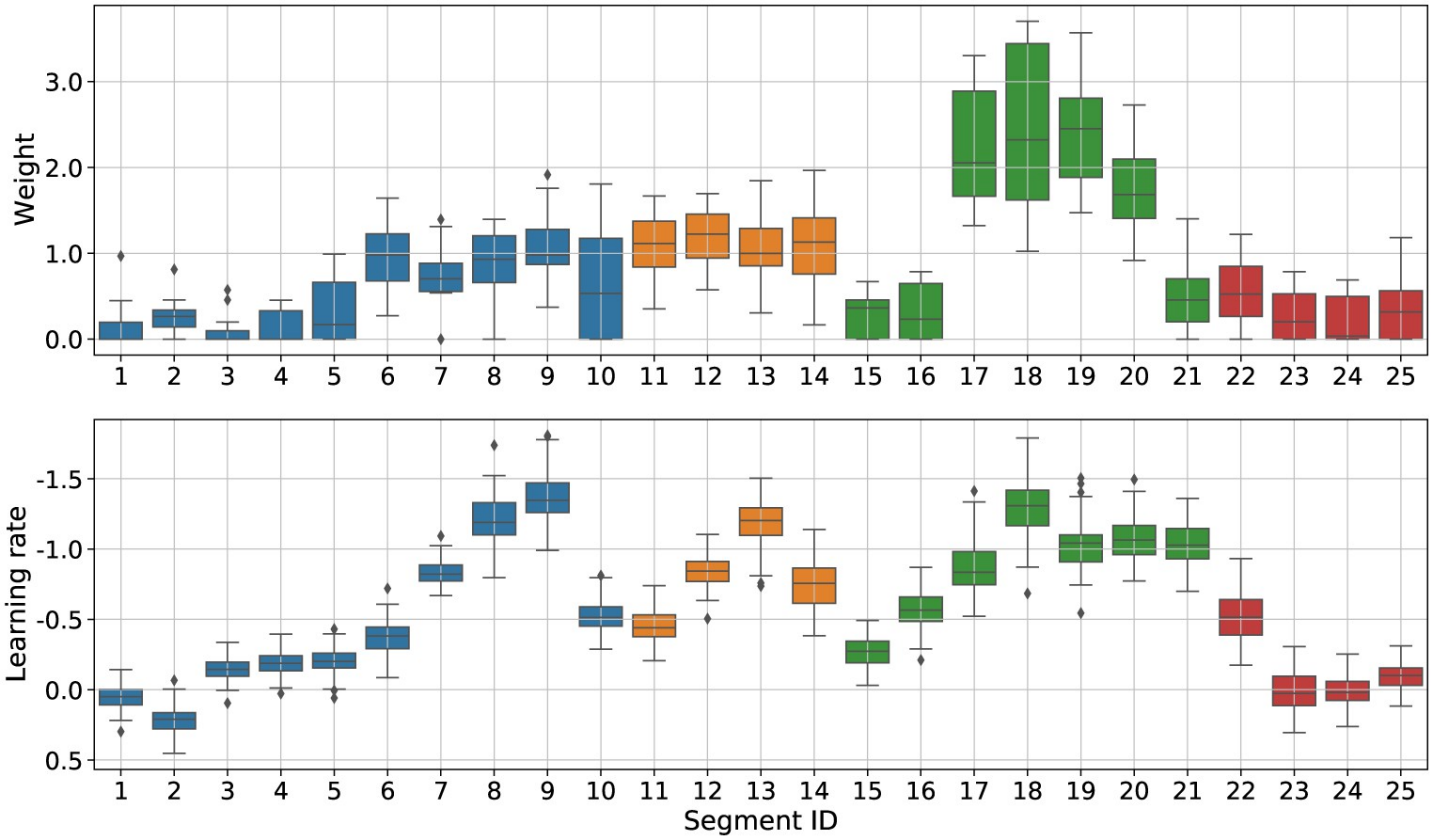
Per-segment learning rate, correlation and optimisation weights. Colours are indicative of the four template’s phrase defined on Fig 2.

The optimised weights for *N* = 25 are depicted in Fig 7, top panel. We ran an ANOVA on the optimised weights with Segment as the independent variable. We found a significant main effect (*F*_24,375_ = 41.21, *p* < 0.001, $\eta_{p}^{2}=0.72$). Post-hoc tests with Holm corrections highlighted several differences. In particular, one group of segments stands out from the others, and whose weights reached significantly higher values than the rest. This group is comprised of segments {17, 18, 19, 20}. Interestingly, the segments are adjacent and occurring at a specific moment of the gesture sequence (see Fig 2), indicating where raters temporally focused their attention in the rating process.

We repeated a similar analysis to the previous section, investigating the potential relationships between the optimised weights and learning rates per segment. Partial DTW contributions were computed per segment as the sum of pairwise distances on the portion of aligned paths described by each segment. These contributions were then ordered by performance time and learning rates were extracted from a fitted exponential model (see DTW errors and learning rates per temporal segment in S1 Apparatus). The learning rates are reported below the optimisation weights in Fig 7. We found a significant linear relationships between the means of optimised weights and learning rates (*r*(25) = −0.70, *p* < 0.001). By comparing adjacent segments with t-tests, we identified three main regions of motor improvement located in segments [7–9], [12–14] and [16–21]. The beginning and end of the movement showed the smallest learning rates. Interestingly, while three main regions exhibited some learning progress, only the last one was favoured by the ratings. This suggests that, although learning occurred predominantly at three different times during the movement, the technique revealed that annotators ultimately placed more importance on the last of these three occurrences.

## Discussion

In this paper, we proposed a method that learns a similarity metric which matches human relative similarity ratings of pairs of movements with respect to a template.

First, we found that the relationship between the DTW similarity metric with all movement features and continuous human ratings can be approximated as a linear function (*R* = 0.76), with respect to the annotated database we considered. In other words, linear and rotational accelerations, positions and its derivatives conveyed useful information to characterise the movement. This result seems to indicate that raters referred to a multidimensional complex of information. In a higher dimensional space, metric learning could allow to find a lower dimensional manifold where the ratio between information and noise is improved [26]. This also echoes other results with similar data where feature selection for time warping of long movement sequences was not found to be beneficial [27] and significantly differ from other work involving data with larger feature spaces, such as skeleton data used by [7, 8].

Second, we found that this correlation could be optimised by weighting either movement features or time segments. Statistically significant improvements were found in both cases as compared to baseline even though the improvements were rather small: + 1.6% increase from 0.76 compared to 0.77 for the spatial case; + 4.8% increase from 0.76 compared to 0.79 for the temporal case. While we used diagonal covariance matrices in the Mahalanobis distance to limit the number of parameters to learn, a full covariance matrix should technically improve the correlation value, and was confirmed by preliminary testing. However, such approach has two main drawbacks. First, it would require the collection of a larger dataset to mitigate the risk of overfitting, which was challenging in our case of human-provided ratings. Second, the learned metric would also be more difficult to interpret than in the case of the diagonal matrix which simply amounts to weighting each dimension of the feature space. As a matter of fact, interpretabilty represents an important feature of our method and contrasts with other works in metric learning wherein classification performance improvements do not necessarily need to be explained [13, 14].

Regarding metric interpretation, the optimised weights provide valuable information about important features that the raters perceived to judge the movement similarity. For the spatial case, the three largest weights (*acc*_*x*_, *gyr*_*y*_ and *p*1_*x*_) point towards the type of movement parameters that the raters seemed to focus on: acceleration in the horizontal plane (typically occurring after the vertical strokes), rotation of the wrist (appearing in the preparation and during the strokes), and velocities in the vertical axis (typically during strokes). In contrast, the optimised weights on features along the *z* axis are among the lowest (e.g. *p*0_*z*_ = 0.015). Since the movement is executed mostly in the X-Y plane, this validates the fact that the learned metrics extract meaningful information from the data. This is in line with previous work using input weights learning as a way to analyse human perception [6]. In this paper, we went further by exploring also the temporal dimension. For the temporal case, the very beginning and end show lower weight values meaning that these movement segments (segments 1–5, 21–25) do not provide meaningful information in assessing movement similarity. This is also the case during the two transition moments (segments 10–11 and 14–15). In contrast, we found that the most important segments for assessing movement similarity were located in the third phrase (segments 17–20, in green). Interestingly, this is the most complex phrase to perform, requiring to articulate several circle arcs in different directions. Typically, the participants made several mistakes such as inverting the movement directions, or missing the starting point of a rotation or the number of rotations.

More importantly, in addition to having found strong correlations between learned weights and motor learning rates in both spatial (*r*(15) = −0.62) and temporal (*r*(25) = −0.70) cases, the analysis of their relationship offer interesting opportunities for interpretation. In the spatial case, we found that the weight for the dimension p0z (position in depth) is close to 0, whereas associated learning rate suggest that some progress was also made by participants on this axis. Likewise, in the temporal case, it appears that motor learning occurs in segments following the strokes (segments [7–9] in phrase 1, segments [12–14] in phrase 2, and segments [17–21] in phrase 3) but only the last segment is favoured through optimisation. This evidence shows that the learned metric is able to highlight what caught the attention of the raters when assessing the similarity between movements. Therefore, the method goes one step further than the state of the art on metric learning applied to motion perception [9, 11] as it highlights where motor learning occurs as well as where human raters perceive improvements in movement execution. In addition, this result is based on averaged ratings across raters, which suggests that raters were consistent with respect to the spatial and temporal foci of attention in movement similarity assessment. However, nothing prevents using the method considering a single rater which will highlight idiosyncratic choices in movement similarity assessment.

This paper showed that metric learning is a promising approach as a tool for probing how humans perceived motion similarity and progress in movement execution. In this study we tested our method with one dataset taken from previous work, which included a specific sensor configuration. Our method could, however, be used in future research to draw broader conclusions about the links between movement perception and motor learning, by investigating to which extent movement characteristics might generalise across different datasets.

## Conclusion

We propose a method that uses metric learning to obtain a similarity metric to match human annotators. This is among the first attempts to use metric learning in the context of motor skill learning, especially considering complex movement sequences. We show that the method is effective in providing information on salient movement features and temporal moments that human annotators focused on. Such information can be corroborated with motor learning processes in our case. Further studies can explore these findings in order to clarify criteria used in movement annotation that are known to be difficult to formalise.

Our method is generic and could be applied in other scenarios. For example, it could be used with other algorithms other than DTW, such as probabilistic models (e.g. Hidden Markov Models [28]) or Neural Networks [29]. Importantly, we believe that this is an important first step toward building interactive approaches of annotating complex movement, where similarity metrics could be adapted using human ratings.

## Supporting information

S1 Apparatus (PDF)
