# Supplementary material for: Describing movement learning using metric learning
Source: PLoS One. 2023 Feb 3;18(2):e0272509. doi: 10.1371/journal.pone.0272509 (PMC9897515; doi:10.1371/journal.pone.0272509)
Supplement: S1 Apparatus — (PDF) [file pone.0272509.s001.pdf]

Supporting information

S1 Apparatus. This document (Figure 8) shows the annotation interface that was presented to judges. Its simplicity is quite different than what can be proposed for expert annotations.

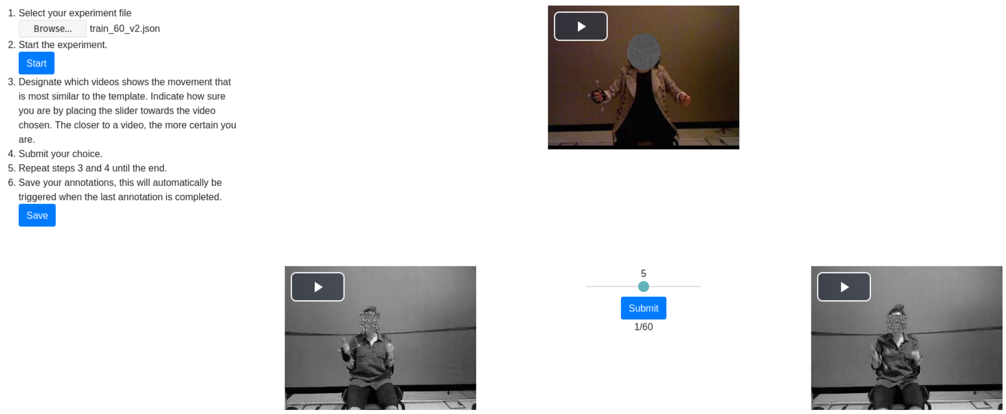

Fig 8. Screenshot of the interface that was presented to annotators during the experiment. The video of the gesture template T is presented on the top and can be watched at any moment. Directly below, the videos of the current pair of motions A and A' are displayed, on the left and right, respectively. In between, a slider serves the purpose of recording the relative measure of motion similarity to the template. In case the distances are similar, the slider should be left in the middle. If A is closer to T than A', then the slider should be moved to the left in proportion to the certainty of the rater. The videos were anonymised. A HOG filter was run on each video frame to identify faces. The region around these was then scrambled to remove any recognisable features.

S2 DTW errors and learning rates per movement feature. DTW errors per movement feature were extracted as follows. The baseline DTW alignement with all 15 features was computed, between performed movements and the template. For each feature, the error was accumulated along the alignment path, yielding one value per dimension (15 in total). These partial costs were re-ordered according to their place in the learning schedule, displayed in figure 9, from which learning rates can be computed, per movement features.

S3 DTW errors and learning rates per temporal segment. DTW errors per temporal segments were extracted as follows. The baseline DTW alignement with all 15 features was computed, between performed movements and the template. N segments

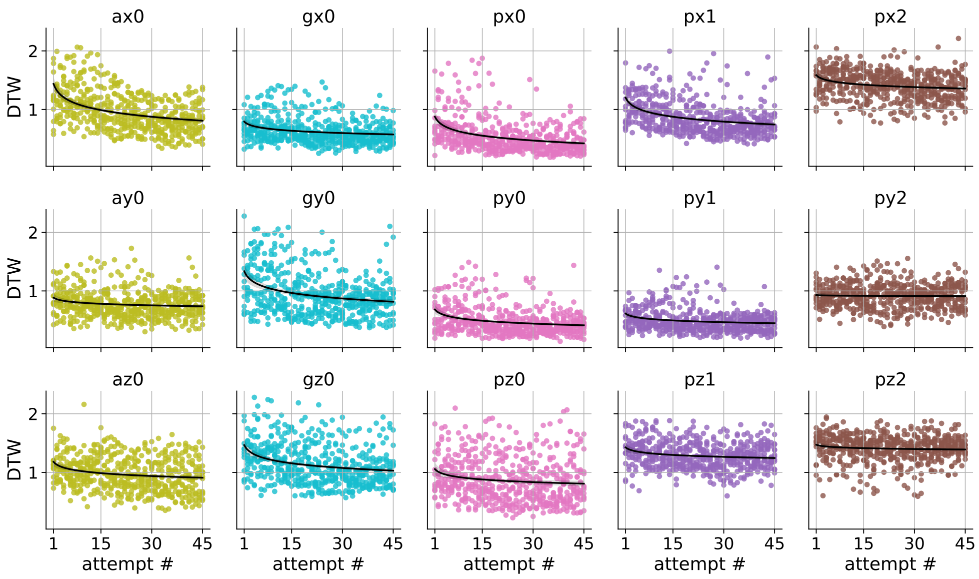

Fig 9. DTW errors as a function of movement feature (title) and session in the learning schedule derived from the DTW alignement including all 15 festures in the point-wise distance. Learning rates are computed from the fit of an exponential model represented by a black line. Colours are unique per data type (acc, gyr, p0, p1, p2).

were then defined on the template. The partial contribution of the alignement cost to the total DTW was then computed per temporal segment, yielding one value per temporal segment (N in total). These partial costs were re-ordered according to their place in the learning schedule, displayed in figure 10, from which learning rates can be computed, per temporal segments.

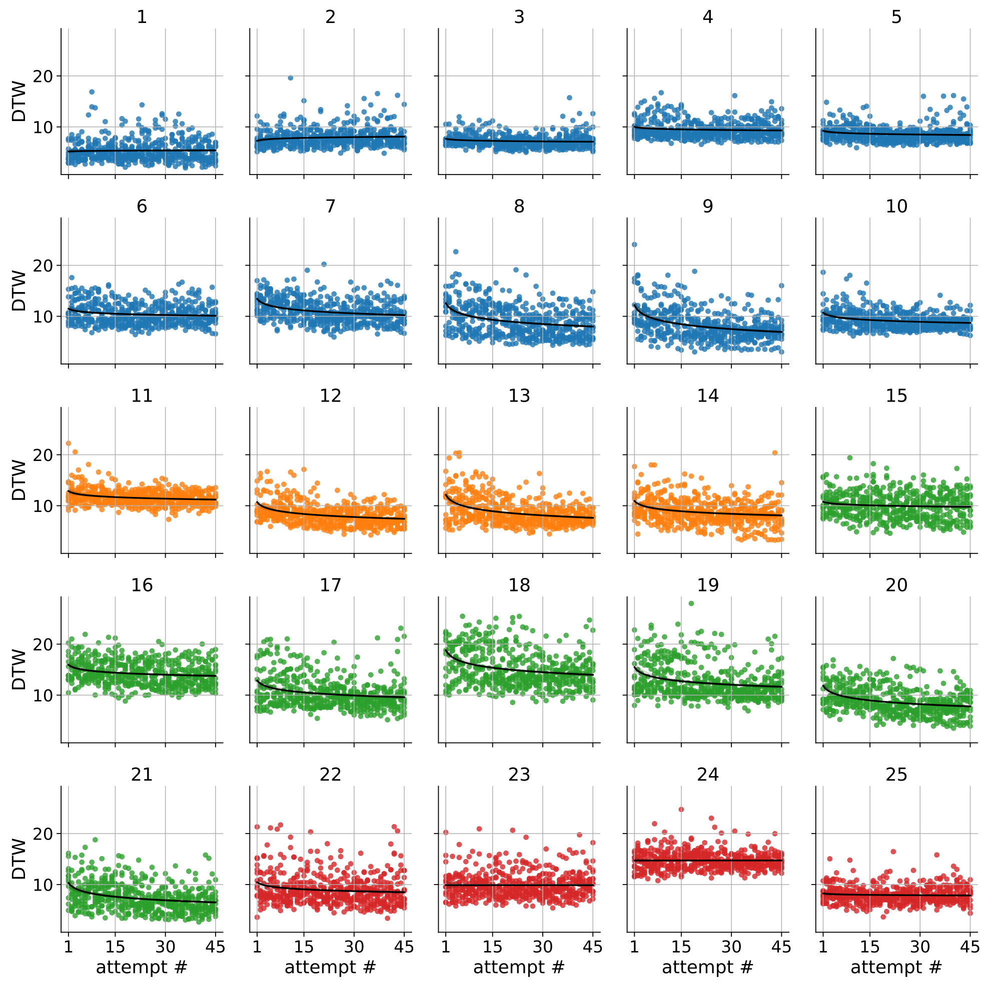

Fig 10. DTW errors as a function of temporal segment (title) and attempt in the learning schedule. Learning rates are computed from the fit of an exponential model represented by a black line. Colours are unique per phrase. The three training sessions covered attempts 1-15, 16-30 and 31-45, respectively.
